# The effect of productive vocabulary knowledge on second language comprehension

**DOI:** 10.3389/fpsyg.2023.1049885

**Published:** 2023-04-14

**Authors:** Takara Kenza Allal-Sumoto, Kiyofumi Miyoshi, Hiroaki Mizuhara

**Affiliations:** Graduate School of Informatics, Kyoto University, Kyoto, Japan

**Keywords:** second language vocabulary processing, comprehensive knowledge, productive knowledge, formulaic sequences, generalized linear mixed model, response time

## Abstract

Second language learners tend to focus more on learning the meaning of vocabulary than on how to use it in their speech and writing. Although comprehensive vocabulary knowledge is necessary for understanding sentences, productive vocabulary knowledge may also have a positive impact on sentence comprehension. Most studies examining the relationship between production and comprehension have compared these abilities between participants or evaluated unrelated criteria between tasks, which may be insufficient for examining the direct effects of productive knowledge on sentence comprehension. Our study investigates changes in sentence comprehension speed during listening, and we used a within-subjects comparison to examine the effect of productive vocabulary knowledge or the lack thereof. We applied generalized linear mixed models to investigate productive vocabulary knowledge effects by partialing out other influential factors, such as confidence, frequency of target words, stimulus duration, and individual differences. The results showed that the sentences with a producible phrase were processed faster than the sentences that included phrases with only comprehensive knowledge or no comprehension. The effect of productive vocabulary knowledge on the speed of sentence comprehension was directly examined with a within-subject comparison, and its contribution was clearly found. This study emphasizes the value of productive vocabulary knowledge acquisition in enhancing the speed of sentence comprehension.

## 1. Introduction

Fast language comprehension is a crucial ability in learning and using a language, especially during the online processing that occurs in listening and speaking. Developing efficient listening comprehension is important, as adults spend 40–50% of their communication time listening, whereas reading is estimated to occupy only 11–16% (Gilman and Moody, 1984). Given that people cannot control the speed of input during listening, like they can during reading, they need rapid and effortless comprehension to keep pace with the flow of input. For second languages, a wide range of proficiency levels exist for comprehension. For example, some second language learners can comprehend a sentence by breaking down each word and translating it into their first language, while others may understand a sentence automatically without translation, just like native speakers (Upton and Lee-Thompson, 2001).

This difference in comprehension proficiency might be related to the presence or absence of productive vocabulary knowledge. In second language acquisition research, it is widely accepted that comprehensive vocabulary knowledge is acquired before productive knowledge is acquired (Read, 2000), and according to Segalowitz and Hulstijn (2005), processing speed quickens as vocabulary proficiency develops. Although there is no doubt that acquiring comprehensive vocabulary knowledge is essential for sentence comprehension, the effects of productive knowledge may also have an impact on sentence comprehension.

In previous studies, cross-sectional effects have been identified, specifically the interactions between language comprehension and production abilities. Test scores for general vocabulary production and comprehension have also been positively correlated with reading and listening comprehension (Golkar and Yamini, 2007; Li and Zhang, 2019). While these findings are significant for understanding across-subject associations regarding these abilities, the direct effects of productive and receptive vocabulary knowledge on sentence comprehension cannot be ascertained from these results. The reason is that that study did not use the same target vocabularies to measure proficiency in vocabulary production, vocabulary comprehension, and sentence comprehension. For example, Golkar and Yamini (2007) used the score of the Vocabulary Level Test (Schmitt et al., 2001) for comprehensive vocabulary knowledge, the Productive Version of the Vocabulary Levels Test (Laufer and Nation, 1995) for productive vocabulary knowledge, and the Test of English as a Foreign Language (TOEFL) for reading proficiency. In that study, the vocabularies were not consistent throughout all the measurements.

In terms of level of vocabulary knowledge, sharing the same target words in both productive and comprehensive vocabulary knowledge measurements is an effective way to investigate a learner’s knowledge level for each vocabulary—that is, whether a learner has both productive and comprehensive knowledge of the target word, has only comprehensive knowledge, or cannot comprehend the target word at all. On this premise, we designed the sentences in our comprehension test to contain target words of different vocabulary levels, the effects of which were directly examined through within-subject comparisons. Understanding the effects of varying vocabulary knowledge on sentence comprehension may lead to concrete pedagogical suggestions and an emphasis on productive vocabulary knowledge acquisition.

The aim of this study was to demonstrate the importance of productive vocabulary learning by examining the effects of productive vocabulary knowledge on the speed of sentence listening comprehension. To investigate this effect, we applied a formulaic sequence, which is “a phenomenon that encompasses various types of word string which appear to be stored and retrieved whole from memory” (Wray and Perkins, 2000, p. 1). This process might lead to more rapid processing if learners acquired formulaic sequences in chunks and process them as one word rather than as a series of individuals words. In addition, although these high-constraint phrases may be processed more slowly and lexical links may be weaker in a second language than in a first language, processing speed might be enhanced as second language proficiency increases (Ito and Pickering, 2021). Therefore, we hypothesized that the speed of sentence comprehension would change according to the learner’s productive vocabulary knowledge, as the presence or absence of productive knowledge is a reflection of vocabulary knowledge development (Read, 2000; González-Fernández and Schmitt, 2020).

Other influential factors in the speed of second language sentence comprehension might exist, such as confidence, frequency of target words, and stimulus duration. For example, Shen and Jiang (2013) have claimed that learners’ confidence may speed up language comprehension. Therefore, we tested the contribution of vocabulary knowledge to sentence comprehension using a generalized linear mixed model (GLMM) to partial out the effects of other influential factors.

Individual differences among second language learners might also reflect a mixture of unmeasured variables, such as the starting age of second language acquisition, learning methodologies, personalities, and cognitive styles (Fan, 2000; González-Fernández and Schmitt, 2015; Yang et al., 2015). These individual differences might affect the variables of present interest (vocabulary knowledge, confidence, frequency of target words, and stimulus duration). Individual differences are miscellaneous. Therefore, we considered individual differences that are unable to be measured directly in this study in the GLMM analysis as a random intercept and random slopes to enhance the soundness of within-subject comparison analysis. We were able to demonstrate a relationship between productive vocabulary knowledge and sentence comprehension and identify the extent to which productive vocabulary knowledge improves the speed of comprehension.

## 2. Materials and methods

### 2.1. Participants

In this study, 37 undergraduate and graduate students at Kyoto University (17 males, 20 females) participated. Initially, the sample size was determined by a power analysis for a one-way repeated measure ANOVA, but the main analysis was conducted by GLMM. The results of the one-way repeated measure ANOVA are reported in Supplementary material. Because we applied the novel approach of a within-subject comparison, the expected effect size could not be found from previous studies. Therefore, as a practical choice, a medium effect size with a one-way repeated measure ANOVA was calculated for knowledge only, which was the focus of this study. The rest of the parameters followed the default settings of GPower (Version 3.1.9.6). The required number of participants for the statistical analysis was 28 participants, and this was decided before the experiment *via* a power analysis using GPower with the following criteria: *α* = 0.05, 1-β = 0.8, effect size *f* = 0.25, corr among rep measures = 0.5, and the number of measurements = 3 (productive knowledge, comprehensive knowledge only, and noncomprehension).

The mean age of the participants was 22.5 ± 1.57 (range: 20–26 years). The participants’ first language was Japanese, and they had attained one or more of the following English proficiency test scores within 2 years of participation: over 600 on the Test of English for International Communication (TOEIC), over 503 on the TOEFL Institutional Testing Program (ITP), over 61 on the TOEFL Internet-Based Testing (iBT), or over 5.0 on the International English Language Testing System (IELTS). All participants were right-handed according to the Edinburgh Handedness Inventory (Oldfield, 1971), and they all received an honorarium. This study was approved by the ethics committee of the Kyoto University Psychological Science Unit (approval number: 1-P-2).

### 2.2. Stimuli

We adopted a formulaic sequence as the target for the tasks. We selected adverb and adjective phrases of three or four words that can be placed at the end of sentences as formulaic sequences. Certain phrases were omitted from the target selection to control the difficulty of comprehension and retrieval: phrases that have word repetition (e.g., “again and again”), phrases that consist of antonyms (e.g., “sooner or later”), and phrases that include possessive pronouns depending on a sentence (e.g., “above one’s head”). We referred to two corpora to extract a wide frequency range of phrases (range: 7–36,475 appearances in the Corpus of Contemporary American English and 2–6,875 appearances in the British National Corpus), and we selected 200 phrases.

**Figure 1:**
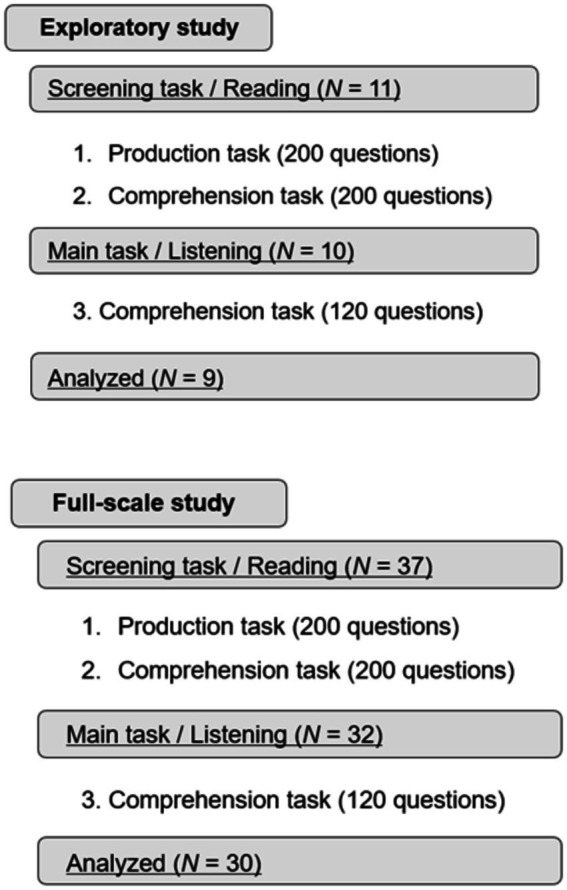
The structure of the full experiment. The top reflects the exploratory study and the bottom reflects the full-scale study. The screening phase included a translation task to measure the productive and comprehensive knowledge of various phrases. All the stimuli in the screening task were presented visually. The main task included a listening comprehension task. All the stimuli in the main task were presented in an auditory format.

The experiment consisted of two parts: (1) a screening task with a production task and a comprehension task, and (2) the main task with a comprehension task (Figure 1). To show different sentences for each task, we chose three different sentences for each selected phrase (200 each, total 600) from example sentences from several dictionaries, such as the Cambridge Learner’s Dictionary, the Oxford Learner’s Dictionary, and the Collins Online Dictionary. Learner’s dictionaries are for intermediate to upper-intermediate learners, and they provide example sentences with easier words and grammatical structures than other general dictionaries. To further balance the difficulty of sentence comprehension, we edited some sentences from the dictionaries so that all sentences consisted of eight to 10 words. All questions in the main task were presented in auditory format, and the English sentences were generated by Google’s text-to-speech function with a reading speed of 150 wpm, the average speech speed of native speakers (Wang, 2021).

### 2.3. Task procedure

**Figure 2:**
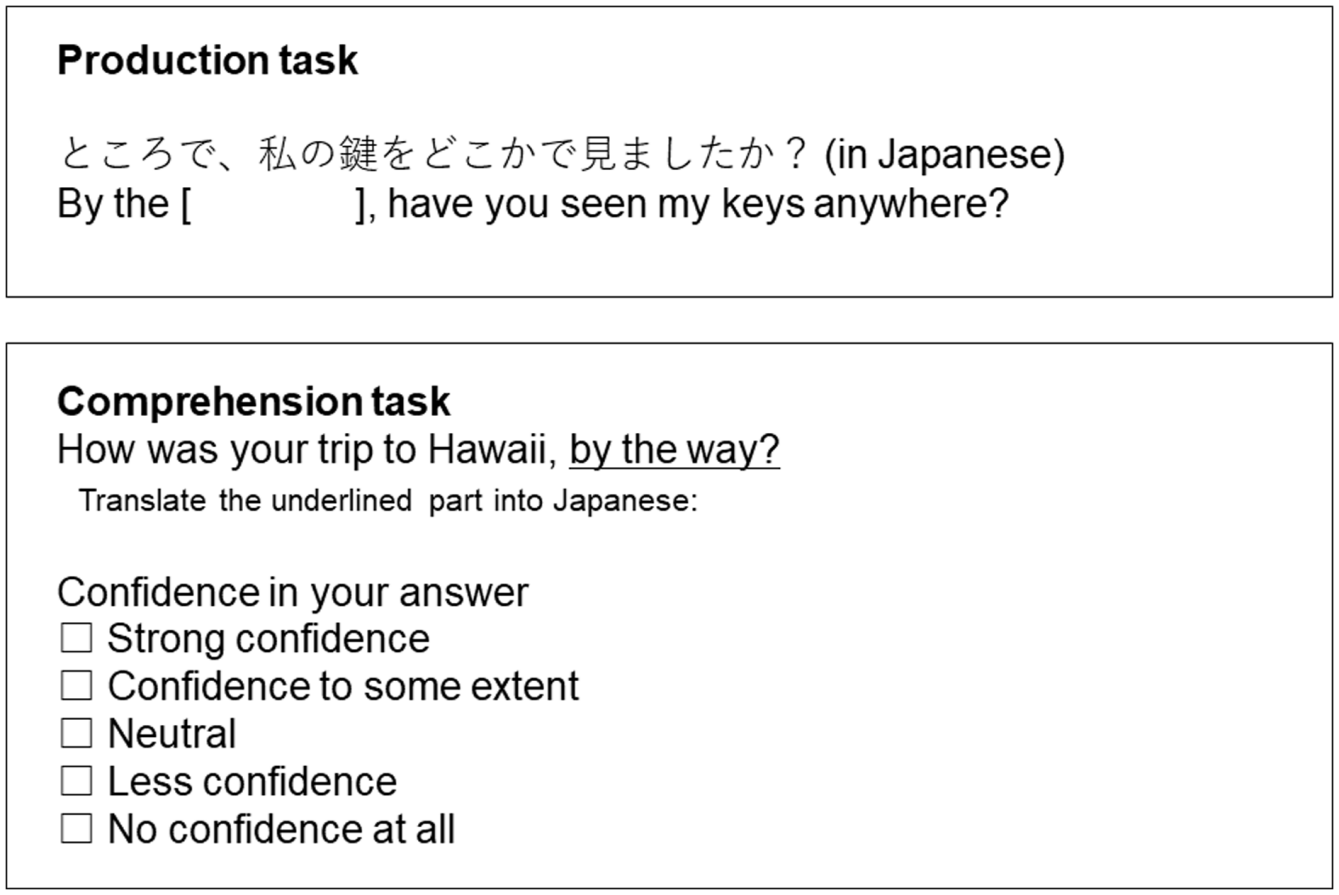
An example question from the screening task. The top includes an example question from the production task. The Japanese sentence corresponds to the English sentence. The bottom includes an example question from the comprehension task. The underlined word is the target phrase.

The screening task was conducted for two purposes: to obtain information on participants’ knowledge of each phrase and to select participants for the main task. The screening was conducted on Google Forms and involved two sets of 100 questions for each of the production and comprehension tasks, so the participants answered a total of 400 questions. In the production task, to examine whether participants could produce the target phrases, we presented pairs that had one complete Japanese sentence and one English sentence in text form on the screen. The last word of a phrase in the English sentence was missing, and the participants were asked to type the missing word on the Google form. The accuracy of spelling was not strictly judged, and near-accurate spellings were treated as correct answers. Next, in the comprehension task, we presented a complete English sentence with the target phrase underlined. Participants were required to write Japanese translations only for the underlined phrase to determine the participants’ comprehensive knowledge of the phrases. Participants were also asked to rate their confidence in understanding the underlined phrases on a Likert scale (5: strong confidence, 4: confidence to some extent, 3: neutral, 2: less confidence, and 1: no confidence at all; Figure 2). There was no time limit, but we advised the participants to answer 100 questions in 30 min.

Using the screening task results, we divided the phrases into three knowledge groups: phrases that participants could both produce and comprehend, phrases that participants could comprehend but could not produce, and phrases that participants could neither comprehend nor produce. We omitted the phrases that participants could produce in English but could not understand the meaning of, as we assumed that this was an accidental occurrence rather than a true indication of vocabulary development. According to the central limit theorem, it is often said that a sample size of at least 30 would assume an approximately normal distribution for the sample mean (Smith and Wells, 2006). In addition, a 10–30% larger sample would be required for missing data as backup (Shuai et al., 2012). Therefore, we decided that only the participants with knowledge of at least 30 or more target phrases in each of the three different vocabulary knowledge from the screening task should proceed to the main task. The main task consisted of 120 questions, of which at least 30 were selected from each of the three types of vocabulary knowledge, with additional questions selected depending on the results of each individual participant’s screening task.

**Figure 3:**
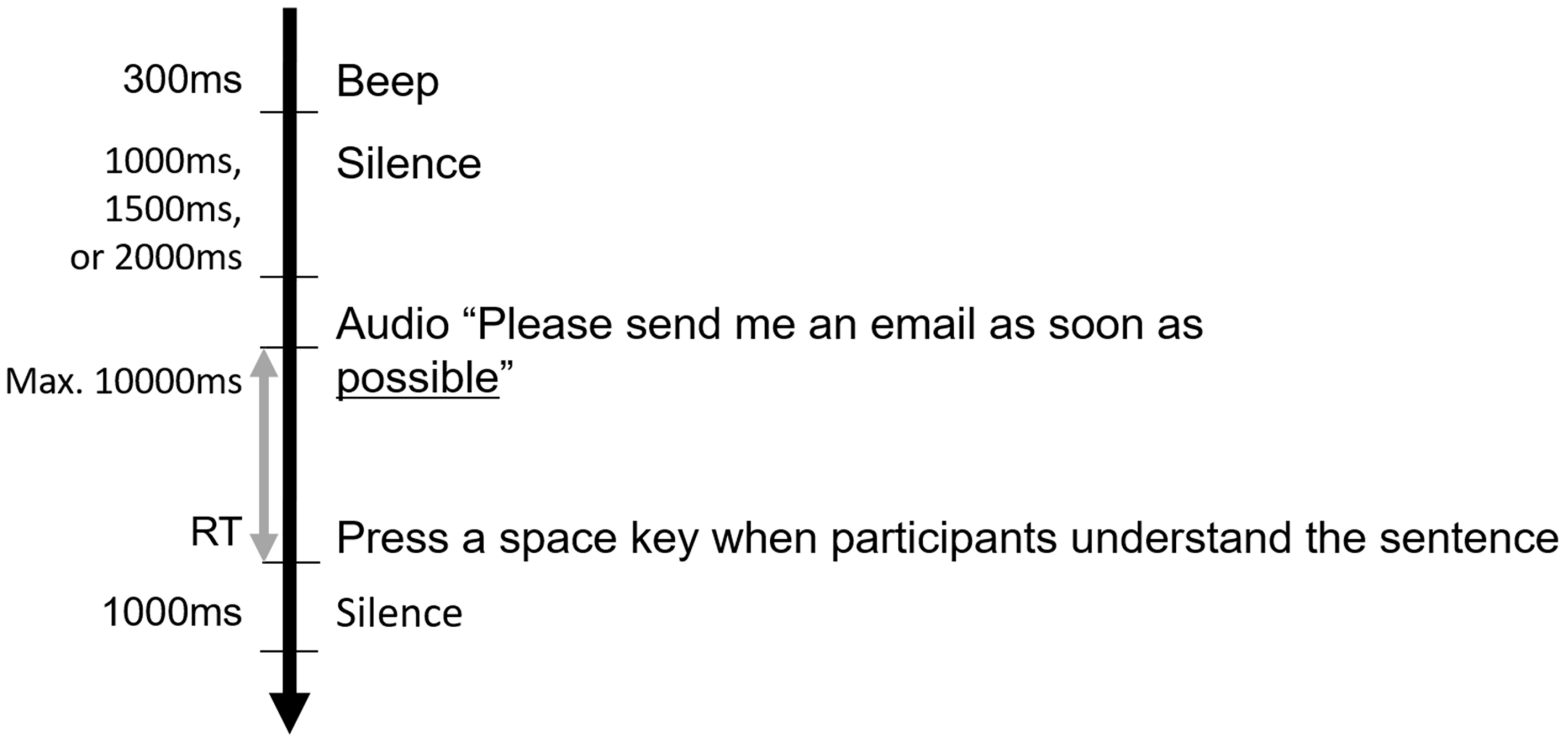
The flow of comprehension tasks in the main task. The black arrow indicates the flow of one trial. The times on the left side indicate the duration of the activities. Each trial began with a beep sound and finished with silence that lasted for 1,000 ms. RT (represented by the gray arrow) indicates the response time for sentence comprehension. Each sentence was played once. The underlined word is the last word of the target phrase.

The main task was held at least 1 week after the screening task to minimize the effect of recent memory. The stimuli in the main task were set specifically for each participant based on the results of the screening task, and the participants were not informed of their knowledge levels for each word. The main task, which was conducted online with Psytoolkit (Stoet, 2010, 2017), consisted of two sets of 60 trials, so the participants answered 120 questions. All the stimuli except for the procedure explanation were presented in audio format. First, the participants adjusted the sound volume and answered five practice questions. In the comprehension task, each trial began with a 300 ms beep, followed by a silent period of 1,000, 1,500, or 2,000 ms, which was applied randomly (Figure 3). Next, an English sentence was presented in which the last three or four words consisted of the target phrase. The participants were asked to press the space key as soon as they comprehended the sentence. Response time was defined as the time between the onset of the last word and when the participants pressed the space key. Participants were informed that they could even press the space key as the audio was playing. Thus, when participants pressed the space key during the audio stimulus, the response time was recorded as a negative value. All the measured times were adjusted to positive values by adding 2,620 ms, since −2,618 ms was the shortest response time. Nonresponse trials (*M* ± *SE* = 19.17 ± 2.00%) were omitted from the data analysis. Since we aimed to compare the response times for sentence comprehension, which included the target phrases of different within-subject vocabulary knowledge, the response times in the main task were sorted according to the screening task grouping. For statistical analysis, we analyzed the data of 30 participants with more than 10 target phrases from each of the three types of vocabulary knowledge.

### 2.4. Statistical analysis

An exploratory study was conducted first with the 11 participants who were different from and not included in the 37 participants mentioned in section 2.1 (Participants). Using the results of the exploratory study, we determined the criteria for participant selection and fixed the analysis pipeline before the full-scale study. In the full-scale study, five participants did not proceed to participate in the main task because of the results of the screening task. As an exception, two participants who had only 29 incomprehensive phrases were allowed to participate in the main task so that we could achieve desirable statistical power. From the main task results, the data from two participants were not included for statistical analysis, as the numbers of their data points did not reach the criteria. We then analyzed the data from 30 participants.

All statistical tests were conducted using R (R Core Team, 2021). To investigate whether the response time remained unchanged according to vocabulary knowledge, we implemented a GLMM using the *lme4* package (Bates et al., 2022) and *multcomp* (Hothorn et al., 2008) in R (R Core Team, 2021) to evaluate the potential effects of different types of vocabulary knowledge (i.e., productive knowledge, comprehensive knowledge only, and noncomprehension) and other variables (confidence, phrase frequency, and stimulation duration) as fixed effects on the speed of sentence comprehension. A random intercept and random slopes for these four variables were considered across participants. In the GLMM analysis, we assumed a gamma distribution for the outcome variable (adjusted response time data). For better model convergence, the adjusted response time, phrase frequency, and stimulus duration were scaled by 1:1,000. If the main effects on the speed of sentence comprehension were found using Type III Wald chi-squared tests, we conducted Tukey tests for multiple comparisons.

## 3. Results

A GLMM analysis was applied to investigate the contributions of each variable—knowledge, confidence, phrase frequency, and stimulus duration—on the speed of sentence listening comprehension. We especially focused on the contribution of knowledge to the speed of comprehension by partialing out the other variables’ effects. We set eight models, as shown in Table 1. Model 1 consisted of knowledge as a fixed effect, a participant-wise random slope of knowledge, and a participant-wise random intercept. Subsequent models included greater numbers of variables. Model 8 was a full model that included knowledge, confidence, phrase frequency, and stimulation duration as fixed effects with participant-wise random slopes for these variables and a participant-wise random intercept.

**Table 1:** Models used for the generalized linear mixed model (GLMM) analyses and the model selection by the Akaike information criterion (AIC) and the Bayesian information criterion (BIC).

Model	AIC	BIC
Model 1: RT = α_0_ + S_0j_ + (β_1_ + S_1j_)X_1_ + ε	9621.947	9681.703
Model 2: RT = α_0_ + S_0j_ + (β_1_ + S_1j_)X_1_ + (β_2_ + S_2j_)X_2_ + ε	9576.485	9666.119
Model 3: RT = α_0_ + S_0j_ + (β_1_ + S_1j_)X_1_ + (β_3_ + S_3j_)X_3_ + ε	9597.005	9686.639
Model 4: RT = α_0_ + S_0j_ + (β_1_ + S_1j_)X_1_ + (β_4_ + S_4j_)X_4_ + ε	9616.650	9706.283
Model 5: RT = α_0_ + S_0j_ + (β_1_ + S_1j_)X_1_ + (β_2_ + S_2j_)X_2_ + (β_3_ + S_3j_)X_3_ + ε	9559.900	9685.387
Model 6: RT = α_0_ + S_0j_ + (β_1_ + S_1j_)X_1_ + (β_2_ + S_2j_)X_2_ + (β_4_ + S_4j_)X_4_ + ε	9574.070	9699.557
Model 7: RT = α_0_ + S_0j_ + (β_1_ + S_1j_)X_1_ + (β_3_ + S_3j_)X_3_ + (β_4_ + S_4j_)X_4_ + ε	9640.229	9765.715
Model 8: RT = α_0_ + S_0j_ + (β_1_ + S_1j_)X_1_ + (β_2_ + S_2j_)X_2_ + (β_3_ + S_3j_)X_3_+ (β_4_ + S_4j_)X_4_ + ε	9555.767	9723.083

RT, response time; X_i_, independent variable (X_1_, Knowledge; X_2_, Confidence; X_3_, Stimulus duration; X_4_, Phrase frequency); α_0_, fixed intercept; S_0j_, random intercept for jth participant; β_i_, fixed slope for ith independent variable; S_ij_, random slope for ith independent variable for jth participant; ε, residual error.

**Figure 4:**
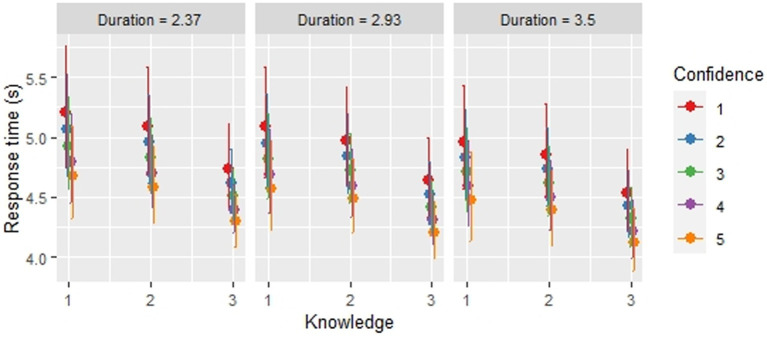
The relationship between sentence comprehension speed, knowledge, confidence and stimulus duration based on GLMM Model 8. In each plot, the point in the middle represents the predicted value of the response time for sentence comprehension at each knowledge level derived from Model 8. The lines indicate the 95% confidence intervals for the predicted values. The numbers at the bottom indicate the level of knowledge: 3. Producible, 2. Only comprehensible, and 1. Not comprehensible. The points with different colors show each confidence level from 1 to 5. The graph in the middle shows the result with the mean of stimulus duration. The left and rights graphs are based on the mean of stimulus duration with −1 and + 1 standard deviations. Frequency is not included in the graphs, as it did not significantly affect sentence comprehension speed.

First, in accordance with the work of Barr et al. (2013), we conducted statistical tests based on the full model (Model 8) (Figure 4). The result from the ANOVA Type III Wald chi-squared test revealed, a significant main effect of knowledge [χ^2^(2) = 14.81, *p* < 0.001], confidence [χ^2^(1) = 5.76, *p* = 0.002], and stimulus duration [χ^2^(1) = 6.84, *p* < 0.001] on the speed of sentence comprehension, but no significant effect of phrase frequency [χ^2^(1) = 3.07, *p* = 0.080]. Multiple comparisons made using a Tukey test revealed that the sentences with a producible phrase were processed faster than the sentences that included phrases with only comprehensive knowledge or no comprehension (productive knowledge – only comprehensive knowledge: *z* = 3.137, *p* < 0.001; productive knowledge – no comprehension: *z* = 3.616, *p* < 0.005; only comprehensive knowledge – no comprehension: *z* = 0.923, *p* = 0.625).

As for the model comparison, the Akaike information criterion (AIC) selected Model 8, which included knowledge, confidence, phrase frequency, and stimulation duration as fixed effects and all possible random slopes and intercepts across participants. The Bayesian information criterion (BIC) chose Model 2, which consisted of knowledge and confidence as fixed effects with random slopes for these variables and a random intercept across participants (Table 1). It is common for AIC and BIC to select different models, as these two criteria select models based on different indicators. Therefore, the most significant point is that all the analyses here signified a reliable contribution of knowledge to the speed of sentence listening comprehension. Thus, these results collectively revealed that knowledge is a significant key variable in the speed of sentence comprehension.

## 4. Discussion

### 4.1. Effects of productive vocabulary knowledge on sentence comprehension

The current experiment shows that sentences including producible phrases were processed significantly faster than sentences including only comprehensible phrases and noncomprehensible phrases for comprehension. This result clearly indicates the positive impact of productive vocabulary knowledge on sentence comprehension. In our study, the difference in response time between the sentences with producible phrases and those with only comprehensible phrases was approximately 430 ms. This difference could have a great impact on sentence listening comprehension because language comprehension requires the multifactor processing of phonemes, vocabulary, grammar, pragmatic meanings, and so on in a short period (Rayner and Clifton, 2009; Thompson and Kielar, 2014). It is said that adults can identify spoken words of their first language within 200 ms on average (Ojima et al., 2011), and the meanings of some written words seem to be accessed even faster, at around 100–200 ms (Davis et al., 2019), implying that a great amount of information could be processed in the span of 430 ms.

In terms of sentence comprehension without productive vocabulary knowledge, the participants could understand some sentences with noncomprehensible phrases in the main task, although the average confidence score for noncomprehensible phrases was 1.08 ± 0.11 (*M* ± *SE*) out of 5, meaning that the participants were not confident at all according to the screening task. This finding may be due to participants ascertaining the meaning from the context. As for the processing of sentences that included only comprehensible phrases, no significant differences existing in comprehension speed for sentences with noncomprehensible phrases, even though the participants knew the meaning of the phrases. While processing sentences with only comprehensible phrases and those with noncomprehensible phrases, the participants might have guessed the meaning of the phrases from the context or translated the phrase into their first language for comprehension (Vandergrift, 2003). In these cases, the sentences with only comprehensible phrases and noncomprehensible phrases might have taken longer to comprehend. These results were able to be demonstrated without the effects of other influential factors, which were partialed out.

### 4.2. Possible mechanisms of productive knowledge effects on comprehension

Rapid sentence comprehension might be explained by the automaticity of semantic processing. Segalowitz and Hulstijn (2005) claimed that vocabulary proficiency development leads to rapid processing. They stated that as vocabulary proficiency develops, various processing changes occur: rapid processing, absence of attentional control, unconscious processing, effortless processing, ballistic processing, gain efficiency, and memory-based processing. As a result, some processing changes overlap, and automatic vocabulary processing occurs (Segalowitz and Hulstijn, 2005). Also, Ito and Pickering (2021) noted that the automaticity of lexical processing in a second language seems to be enhanced as learners’ proficiency develops. In our study, it is possible that the sentences that included a producible phrase were comprehended automatically, lowering comprehension speed. Furthermore, Schmitt (2010) stated that the automaticity of vocabulary processing is beneficial not only for vocabulary processing but also for grammatical and pragmatic processing.

Chunking incoming words into a phrase might be a possible interpretation for accelerated comprehension. Chunking helps increase the efficiency of language processing by lowering the brain’s burden to process language and reducing reaction times (Tang, 2013). In addition, by repeatedly comprehending and producing acquired vocabulary, learners become able to memorize and chunk phrases, which increase their processing speed (Segalowitz and Hulstijn, 2005). The results of the current study may be explained by the participants’ memorization of producible phrases as chunks, since acquired formulaic sequences are memorized as chunks, according to Wray and Perkins (2000). The producible phrases also could have been retrieved as chunks during listening comprehension tasks, which would have positively affected the speed of sentence comprehension.

Another possible interpretation of the acceleration of comprehension might be the prediction effect for comprehension. Pickering and Gambi (2018) claimed that listeners constantly predict what words are coming next during language comprehension; although Martin et al. (2013) found no evidence for the prediction of phonology in second language research, the possibility exists that the theory of prediction in language comprehension can be applied to semantic and syntactic information. According to the theory of prediction-by-production (Pickering and Gambi, 2018), prediction speed can only be as fast as the comprehender’s production system. Producible words and phrases can be predicted by accessing the mental lexicon and retrieving it before the phrases are presented, even in a second language, and this process leads to rapid processing. Since productive knowledge is essential for prediction, the prediction would be limited without productive vocabulary knowledge during sentence comprehension. This may lead to longer response times.

According to the motor theory of speech perception (Liberman et al., 1957; Wilson et al., 2004; Iacoboni, 2008), the motor system is involved in mapping the acoustic signal to a phonetic code, and listening performance can be improved by motor cortex activity at the level of perception. Therefore, the result of faster comprehension in the current study might be explained by motor cortex activity. Even though for the current experiment we cannot completely deny such perceptual effects, the response time lag between processing sentences with producible phrases and the other comprehensible and noncomprehensible phrases would be too large if the cause was only perceptual activity (Rayner and Clifton, 2009; Ojima et al., 2011; Thompson and Kielar, 2014; Davis et al., 2019). Acoustic to phonetic mapping in speech perception would be present in less than 175 ms (Bidelman et al., 2013), so the results showing a 430 ms difference between producible phrases and only comprehensible phrases might reflect cognitive effects, such as memory and chunking.

### 4.3. Limitations

Although the results of the current study show the importance of productive knowledge acquisition for the speed of sentence comprehension, some limitations exist. First, we asked participants about their confidence in phrase comprehension during the screening task and not when response time was measured. Therefore, a mismatch might have occurred between confidence in the screening task, in which the stimulus was presented visually, and the main task, in which the stimulus was presented auditorily. Even though there are some drawbacks to asking participants about their confidence during the screening task, there are also certain benefits. In the screening task, the target phrases were underlined, so participants indicated their level of confidence in comprehending the target phrases, not the whole sentences. Although they might have guessed the meaning of a target phrase from the whole sentence, thereby affecting the level of confidence in comprehension, asking about the confidence levels of only the target phrase and not the sentence might have led to a better prediction of confidence in target phrase comprehension. In addition, asking about confidence in the main task would have made the experiment longer, which might have negatively affected the participants’ power of concentration and the speed of comprehension. For these reasons, we obtained data about confidence during the screening task.

Furthermore, we measured the accuracy of vocabulary knowledge during the screening task rather than during the main task. Although a discrepancy might have existed between comprehension accuracy in the screening and main tasks, the purpose of this study is to examine the effects of already acquired vocabulary knowledge on the speed of sentence listening comprehension. For this reason, the accuracy of listening comprehension is beyond the scope of this project.

Finally, studies by Segalowitz and Segalowitz (1993) and Segalowitz and Hulstijn (2005) have called for caution in interpreting response time. A faster response time itself does not explain whether the cause is simple acceleration by habituation or processing changes. Although examining changes in response time by comparing productive knowledge to the lack thereof would not be sufficient to explain its cause, a clear indication exists that something is happening from the change in response time. To ascertain whether processing changes occur in cases with and without productive vocabulary knowledge during comprehension, we plan to conduct a brain recording experiment.

## 5. Conclusion

This study clearly shows that productive knowledge of second language phrases positively affects the speed of sentence listening comprehension. Second language learners can understand more vocabulary than they are able to use in their second language (Laufer, 2005). These learners tend to focus on learning the meaning of vocabulary rather than the usage and spend more time on input than on output. Although acquiring comprehensive vocabulary knowledge is necessary for sentence comprehension, it is only a minimum requirement. Because rapid and stable vocabulary processing is essential, especially during online communication and conversation, the acquisition of productive vocabulary knowledge is important for enhancing the quality of language comprehension.

## Data availability statement

The datasets presented in this study can be found in online repositories. The names of the repository/repositories and accession number(s) can be found: https://researchbox.org/694.

## Ethics statement

The studies involving human participants were reviewed and approved by the Kyoto University Psychological Science Unit (approval number: 1-P-2). The participants provided their written informed consent to participate in this study.

## Author contributions

TA-S: conceptualization, methodology, formal analysis, investigation, writing, and original draft preparation. KM: methodology, software, writing, and reviewing and editing. HM: conceptualization, methodology, writing, reviewing and editing, and supervision. All authors contributed to the article and approved the submitted version.

## Funding

This work was supported by the JSPS KAKENHI (Grant Nos. 20K20860 and 22H03913).

## Conflict of interest

The authors declare that the research was conducted in the absence of any commercial or financial relationships that could be construed as a potential conflict of interest.

## Publisher’s note

All claims expressed in this article are solely those of the authors and do not necessarily represent those of their affiliated organizations, or those of the publisher, the editors and the reviewers. Any product that may be evaluated in this article, or claim that may be made by its manufacturer, is not guaranteed or endorsed by the publisher.
