# Supplementary material for: The effect of productive vocabulary knowledge on second language comprehension
Source: Front Psychol. 2023 Apr 14;14:1049885. doi: 10.3389/fpsyg.2023.1049885 (PMC10140509; doi:10.3389/fpsyg.2023.1049885)
Supplement: Supplementary file 1 [file Data_Sheet_1.pdf]

Supplementary Material

1 Statistical Analysis

All the statistical tests in this supplement were performed using IBM SPSS Statistics version 27 (IBM Corp., Armonk, NY). Before analyzing the relationship between the speed of sentence comprehension and vocabulary knowledge using conventional ANOVA, all the response time data were logarithmically converted, and the logarithm of the response time was averaged for each participant's vocabulary knowledge. In this study, we chose not to conduct outlier exclusion; according to Ratcliff (1993) and Nicklin and Plonsky (2020), transforming all data, including extreme values, is recommended to reduce the influence of outliers (e.g., log transformation, inverse transformation). To adopt the null hypothesis that response time remains unchanged according to vocabulary knowledge, a one-way repeated measures ANOVA was performed with the different types of knowledge (i.e., productive knowledge, comprehensive knowledge only, and noncomprehension) as the independent variable and the log-transformed response time as the dependent variable. A Bonferroni correction was applied for multiple comparisons in case a significant main effect existed.

Given the possible concerns about a spurious correlation between vocabulary knowledge and the speed of sentence comprehension if vocabulary knowledge is correlated with confidence, we evaluated the average confidence level for each type of vocabulary knowledge. A one-way repeated measures ANOVA was used for the three different levels of knowledge as the independent variable and confidence as the dependent variable.

We also performed a one-way repeated measures ANOVA to investigate the confounding effects on the speed of sentence comprehension caused by the other influential variables. The dependent variable included the three different levels of knowledge, and the independent variables were phrase frequency and stimulus duration.

2 Results

2.1 Association between knowledge levels and the speed of sentence listening comprehension

A one-way repeated measures ANOVA with a Greenhouse–Geisser correction was conducted. Mauchly's test indicated that the assumption of sphericity had been violated, $\chi^2(2) = 6.26$, $p < .05$; therefore, the degrees of freedom were corrected using Greenhouse-Geisser estimates of sphericity, $\varepsilon = .83$. The results showed a statistically significant effect of knowledge on the speed of sentence comprehension, $F(1.666, 48.320) = 47.268$, $\eta^2 = 0.62$, $p < .001$. A paired samples *t*-test with Bonferroni correction between each estimated marginal mean was conducted for multiple comparisons. The results indicated that the sentences with producible phrases were processed faster than those with only comprehensible phrases, $t(29) = 7.316$, $p < .001$, Cohen's $d = 1.336$, and those without comprehensible phrases, $t(29) = 8.185$, $p < .001$, Cohen's $d = 1.494$. The sentences with only comprehensible phrases were processed faster than the sentences with no comprehensible phrases, $t(29) = 3.934$, $p = .001$, Cohen's $d = .718$ (Supplementary Fig. 1).

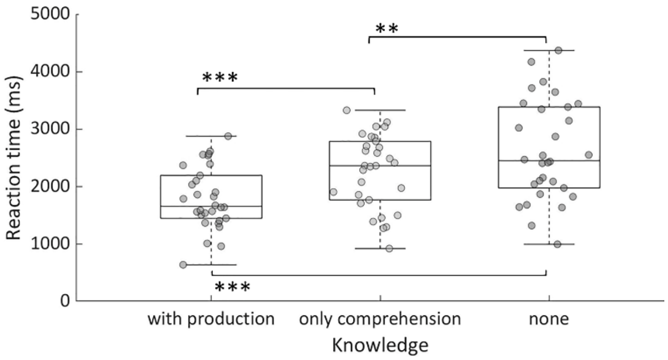

Supplementary Fig. 1 The relationship between knowledge and the speed of sentence comprehension. In the box plots, the line in the middle represents the average response time for sentence comprehension among participants for each knowledge level, and the box encloses the middle of 50% of the data. The top and bottom whiskers extend to the minimum and maximum. The points show the average comprehension speed of each participant ($N = 30$). The asterisks show the significance of the comprehension speed difference between each knowledge level calculated by ANOVA and multiple comparisons with Bonferroni correction with log-transformed response times (***$p < .001$, **$p < .01$, *$p < .05$).

2.2 Association between knowledge levels and the other influential variables: confidence, phrase frequency, and stimulus duration

One-way repeated measure ANOVAs with Greenhouse–Geisser corrections were performed to investigate the associations between knowledge levels and the other influential variables: confidence, phrase frequency, and stimulus duration. For confidence, a significant association existed between knowledge levels and confidence, $F(1.449, 42.027) = 367.297$, $\eta^2 = 0.93$, $p < .001$ (Supplementary Fig. 2a). Significant associations were also found between knowledge levels and phrase frequency, $F(1.249, 36.223) = 576.381$, $\eta^2 = 0.95$, $p < .001$ (Supplementary Fig. 2b), and knowledge levels and stimulus duration, $F(1.656, 48.033) = 43.587$, $\eta^2 = 0.60$, $p < .001$ (Supplementary Fig. 2c). The results of the statistical analysis indicated possible confounding effects of these variables on the speed of sentence comprehension.

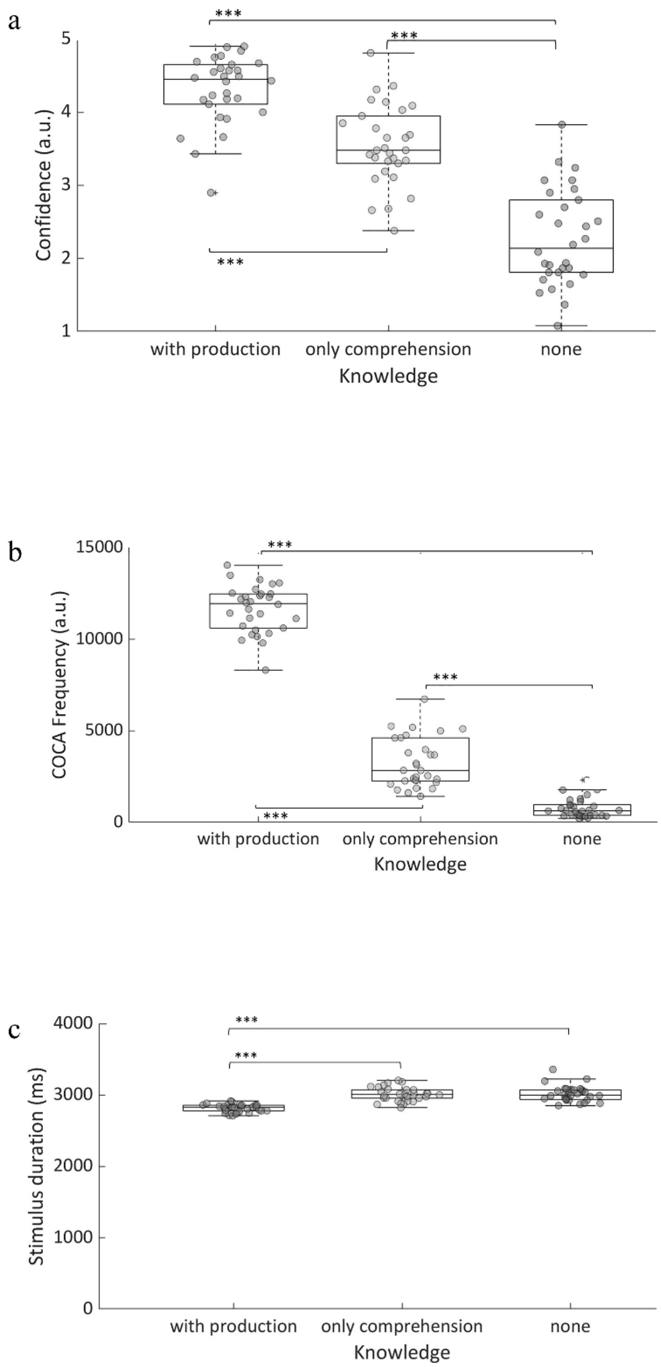

Supplementary Fig. 2 The relationships between knowledge and confidence **(a)**, phrase frequency **(b)**, and stimulus duration **(c)**. In the box plots, the line in the middle represents the average phrase comprehension confidence rate (on a scale of 1 to 5), phrase frequency in the Corpus of Contemporary American English (out of 1,001,610,938 words), and analyzed audio stimulus duration (sentence length excluding the final word) among participants at each knowledge level, respectively. The boxes encloses the middle 50% of the data, and the top and bottom whiskers extend to the minimum and maximum. The points show each participant's average confidence rate, phrase frequency in COCA, and audio stimulus duration, respectively ($N = 30$). The asterisks indicate the significance of the confidence rate difference between each knowledge level calculated by ANOVA and multiple comparisons with Bonferroni correction with confidence rate (***p < .001, **p < .01,

*$p < .05$).

References

Nicklin, C., and Plonsky, L. (2020). Outliers in L2 research in applied linguistics: A synthesis and data re-analysis. *Annual Review of Applied Linguistics* 40, 26–55.

Ratcliff, R. (1993). Methods for dealing with reaction time outliers. *Psychological Bulletin* 114(3), 510–532.
